# Operation-Specific Lexical Consistency Effect in Fronto-Insular-Parietal Network During Word Problem Solving

**DOI:** 10.3389/fnhum.2021.631438

**Published:** 2021-03-10

**Authors:** Chan-Tat Ng, Tzu-Chen Lung, Ting-Ting Chang

**Affiliations:** ^1^Department of Psychology, National Chengchi University, Taipei, Taiwan; ^2^Center for Vital Longevity, School of Behavioral and Brain Sciences, The University of Texas at Dallas, Dallas, TX, United States; ^3^Research Center for Mind, Brain, and Learning, National Chengchi University, Taipei, Taiwan

**Keywords:** mathematical problem solving, numerical processing, word problem, fMRI, prefrontal cortex, posterior parietal cortex, insula, lexical consistency

## Abstract

The practice of mathematical word problem is ubiquitous and thought to impact academic achievement. However, the underlying neural mechanisms are still poorly understood. In this study, we investigate how lexical consistency of word problem description is modulated in adults' brain responses during word problem solution. Using functional magnetic resonance imaging methods, we examined compare word problems that included relational statements, such as “A dumpling costs 9 dollars. A wonton is 2 dollars less than a dumpling. How much does a wonton cost?” and manipulated lexical consistency (consistent: the relational term consistent with the operation to be performed, e.g., more—addition/inconsistent: e.g., less—addition) and problem operation (addition/subtraction). We found a consistency by operation interaction in the widespread fronto-insular-parietal activations, including the anterior insula, dorsoanterior cingulate cortex, middle frontal gyrus, and intraparietal sulcus, such that inconsistent problems engaged stronger activations than consistent problems for addition, whereas the consistency effect was inverse for subtraction. Critically, these results were more salient in the less successful problem solvers than their more successful peers. Our study is the first to demonstrate that lexical consistency effects on arithmetic neural networks are modulated during reading word problem that required distinct arithmetic operations. More broadly, our study has strong potentials to add linkage between neuroscience and education by remediating deficits and enhance instruction design in the school curriculum.

## Introduction

Word problems are a pedagogical practice wherein problems are presented as verbal narratives rather than numerical formulations (Verschaffel et al., 2000). This type of presentation is one of the most common materials in school curricula for teaching students to transfer mathematical knowledge into real-world contexts. Performance on these problems can be predictive of subsequent success in learning higher-level mathematics skills, such as algebra (National Mathematics Advisory Panel, 2008; Fuchs et al., 2012; Powell and Fuchs, 2014). However, most students struggle to solve even the simplest word problems involving whole numbers (Riley et al., 1983; Hegarty et al., 1992; Mullis et al., 2012; Daroczy et al., 2015), although the corresponding numerical notation can be performed proficiently (Cummins et al., 1988). To derive correct word problem answers, problem solvers have to first comprehend problem texts, transfer them into the corresponding mathematical models, and execute the models of the problems (Powell et al., 2015). Therefore, solution difficulty can arise from both problem texts and numerical properties of the solution algorithm (Daroczy et al., 2015). Herein, we attempted to investigate the brain mechanisms of how lexical components of arithmetic word problem description affect problem solution using functional magnetic resonance imaging (fMRI).

In the formation of numerical and reading skills across development, cognitive control processes play the most fundamental role (Geary, 2004; St Clair-Thompson and Gathercole, 2006; Shaywitz and Shaywitz, 2008). Cognitive control refers to the top–down executive mechanisms for voluntarily allocating mental resources based on internal goals and intentions (Miller and Cohen, 2001; Buckner, 2004). Neuroimaging studies have consistently associated brain mechanisms underlying cognitive control with several nodes within the fronto-insular-parietal network, including anterior insula (AI), dorsal anterior cingulate cortex (dACC), and dorsolateral prefrontal cortex (DLPFC), as well as posterior parietal cortex (PPC). The network approach analyses have separated these nodes into two dissociable networks—salience network (SN) and central executive network (CEN) (Seeley et al., 2007; Menon, 2015b). The major components of SN are formed by the AI coupling with dACC (Seeley et al., 2007). This circuit has been associated with subjective salience of external stimuli and contributed to complex cognitive processes including central executive function, as well as affective processes (Menon and Uddin, 2010; Menon, 2015b). The other circuit, CEN, encompasses DLPFC as well as PPC (Seeley et al., 2007; Menon, 2015a). Unlike SN, the CEN and its interconnected nodes are engaged in information retention and manipulation during working memory, constructing problem solution, and goal-oriented decision making (Miller and Cohen, 2001; Petrides, 2005; Rottschy et al., 2012). Converging studies investigating causal interactions between SN and CEN nodes during cognitive control tasks including Stop-Signal, Flanker task, inhibition, and multidigit calculation have revealed that brain signals are initiated from the anterior aspect of the insula toward other nodes within SN and CEN (Kucian et al., 2008; Supekar and Menon, 2012; Cai et al., 2015). Uniquely, the PPC is a highly heterogeneous structure encompassing anatomical subdivisions that appear to play most crucial roles in numerical cognition (Caspers et al., 2006; Choi et al., 2006; Wu et al., 2009; Rosenberg-Lee et al., 2011; Chang et al., 2016). Located anteriorly along the medial bank of PPC, the intraparietal sulcus (IPS) has been associated with representing abstract quantity information and magnitude manipulation (Dehaene et al., 2003; Ansari, 2008; Arsalidou and Taylor, 2011). This entire set of the fronto-insular-parietal network has been implicated in higher-level central executive function and mandatorily associated with numerical cognition, particularly word problem solving (Wu et al., 2009; De Smedt et al., 2011; Chang et al., 2015, 2016, 2019; Menon, 2015c).

Neuroimaging studies have consistently associated the fronto-insular-parietal circuits with arithmetic word problem solving (Prabhakaran et al., 2001; Newman et al., 2011; Zhou et al., 2018; Chang et al., 2019). Chang et al. (2019) found that brain responses toward judging sentences that required one-step mathematical operations (e.g., “There are eight white swans and seven black swans in the pond. There are 15 swans in the pond”) were associated with greater engagement in AI, DLPFC, as well as PPC relative to judgment over parallel narratives without any numerical information (e.g., “There are white swans and black swans in the pond. There are no black swans in the pond”). Prabhakaran et al. (2001) identified that increasing word problem complexity with additional arithmetic operands resulted in increased neural recruitment within the DLPFC. Newman et al. (2011) examined the processing differences between problems presented as verbal narratives, such as “The day before my favorite day is 2 days after Thursday,” and numerical formats (e.g., “4 + 2 = *x* – 1”). They found that relative to the former problem form, the latter relied more on the processing of the IPS. Zhou et al. (2018) found that when solving problems involving straight description and large-quantity calculations, such as “Lucy has 146 marbles. Her brother has 68 marbles. How many more marbles does she have than her brother?” brain responses were associated with greater activations of AI and dACC when compared to solving problems that required verbal reasoning and relatively small-quantity manipulation, such as “Lucy has 90 marbles. Her brother has 60 marbles. How many marbles must she give to him so both of them will have the same amount?” Collectively, the literature demonstrates the fronto-insular-parietal system is engaged by increasing the problem numerical complexity and has suggested a possible role of the problem description form. However, lacking in systematic design and manipulation on lexical components has still left how problem descriptions affect neural mechanisms during reading word problems unclear.

Among the word problem materials, *compare problems* appear to be one of the most challenging curricula (Lewis and Mayer, 1987; Riley and Greeno, 1988; Pape, 2003). This type of problem contains a relational statement comparing the values of two parameters (e.g., a dumpling costs 2 dollars *more* than a wonton). When solving compare problems, students tend to associate the relational term *more* with addition, and *less* with subtraction (Hegarty et al., 1995), such that inconsistent problems in which the relational term is semantically inconsistent with the required arithmetic operation (e.g., less–addition) are more difficult than the consistent ones (Lewis and Mayer, 1987). This finding will hereafter be referred to as the lexical consistency effect. Lewis and Mayer (1987) have demonstrated the lexical consistency effect manifesting more saliently when the relational term is negative (e.g., less, smaller) rather than positive (e.g., more, greater). This is supported by the lexical marking principle, which stated that negative terms are deposited in more complex representation and not easily accessible, and hence said to be “marked,” in contrast to positive terms being “unmarked” (Clark, 1969; Lewis and Mayer, 1987). These have suggested problem solvers directly collect numbers and relational keywords from problem descriptions and bypass the correct problem solution models when solving word problems. It is intriguing to investigate the neural mechanisms of the brain representation of the lexical marking during the sophisticated problem solution process.

**Figure 1:**
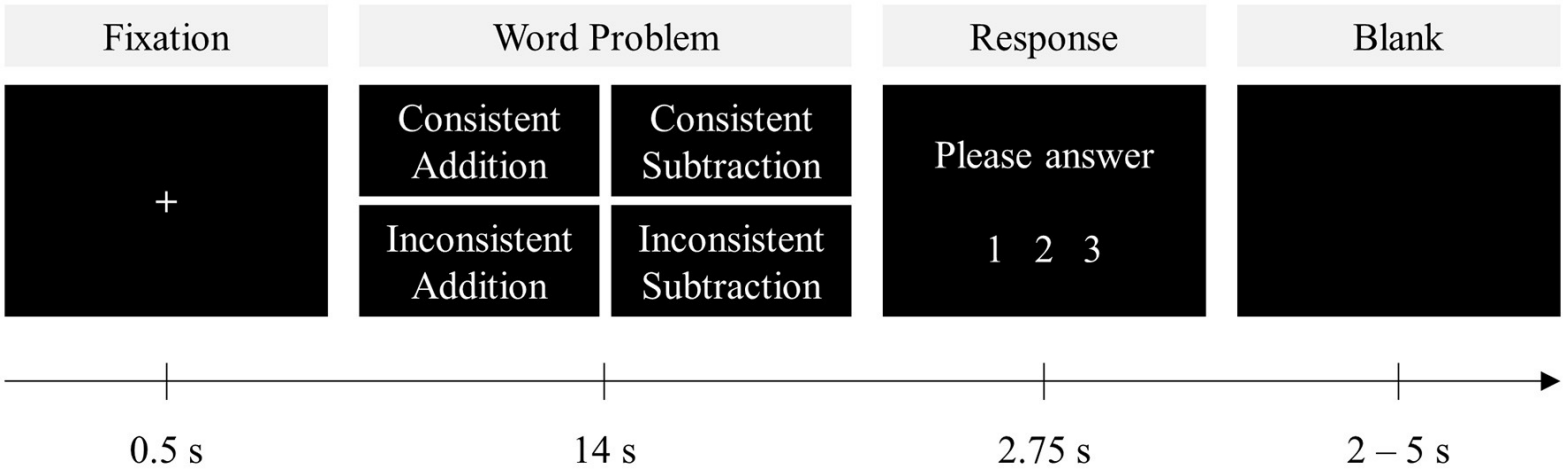
Behavioral paradigm of the present study. Following a fixation sign “+” for 500 ms, a word problem was presented for 14 s. Each problem was orthogonally manipulated and presented in either a consistent or an inconsistent form requiring either addition or subtraction. After the problem presentation, participants were instructed to choose the correct answer from the three provided choices.

In this study, we investigate neural network implications of lexical consistency on arithmetic word problem solving by collecting fMRI data from 36 healthy adults who were proficient at general arithmetic problem solving skills. The critical contrast here was the lexical consistency of *compare problems*. For consistent *compare problems*, the comparison term matched the operation of the correct problem solution (addition—more, subtraction—less), and for the inconsistent problems, they did not (addition—less, subtraction—more). Factors directly manipulated were consistency (consistent/inconsistent) and operation (addition/subtraction; see Table 1 for problem examples for each manipulated condition and Figure 1 for the behavioral paradigm).

**Table 1:** Examples of stimuli for each condition.

**Operation**	**Consistency**	**Example word problem**
Addition	Consistent	There are 4 new books on the bookshelf. There are 5 **more** old books than new books. How many old books are there on the bookshelf?
	Inconsistent	There are 7 penguins on the land. There are 2 **fewer** penguins on the land than in the water. How many penguins are in the water?
Subtraction	Consistent	Kevin has 11 bananas. Stewart has 3 **fewer** bananas than Kevin. How many bananas does Stewart have?
	Inconsistent	The princess has 10 apples. The princess has 4 **more** apples than the witch does. How many apples does the witch have?

*The actual stimuli were presented in traditional Chinese. The relational terms are highlighted in bold type for ease of visualization*.

Based on previous literature that fronto-insular-parietal network was commonly engaged in word problem solutions (Prabhakaran et al., 2001; Newman et al., 2011; Zhou et al., 2018; Chang et al., 2019), we expect that this circuit would vary as a function of the differences between problem conditions. Our central prediction is if problems are solved *via* direct translation, according to the lexical marking principal, problem solvers will focus on the lexical marking of the relational terms whereby the fronto-insular-parietal circuits would show greater activations for *less* (inconsistent addition/consistent subtraction) than *more* (consistent addition/inconsistent subtraction problems) problem conditions; that is, inconsistency problem would engage stronger activations than consistent problem for addition, whereas the consistency effect of subtraction problem would be inverse. In this case, according to Hegarty et al. (1995), unsuccessful problem solvers applied direct-translation strategy and fixated more on problem words and numbers, whereas successful problem solvers tend to construct a mental model and plan the solution based on the model. We therefore further expect that the consistency by operation interaction would be more salient in less proficient problem solvers. An alternative hypothesis is the correct problem mathematical model being constructed regardless of the inherent lexical marking of the problem, and thus fronto-insular-parietal activation will always be a function of the underlying operation.

## Methods

### Participants

Thirty-six healthy adults (18 females and 18 males) were recruited from local educational institutions in Taipei, Taiwan. Because one participant's accuracy of the out-of-scanner task was <3 standard deviations of the group average, and another participant showed reaction time beyond 3 standard deviations of the group average, these two participants were excluded from subsequent analyses, resulting in a final sample of 34 adults (17 females and 17 males) aged between 20.35 and 29.07 [mean = 23.74 (SD = 2.32)]. This sample size was beyond the desired number of 28, as derived from a prospective power estimation using an R package *Superpower* (Lakens and Caldwell, 2019), to reach a power of 1 – β = 0.80 at α = 0.05 based on the average effect size (Cohen *d* = 1.13) found in our previous study assessing brain activations in regions within the fronto-insular-parietal network using adult participants (Chang et al., 2019). All participants were right-handed, with normal or corrected-to-normal vision. None of the participants had reported any history of psychiatric or neurological disorders. Informed written consent was obtained from each of the participants. All participants were treated under the ethical guidelines of the Declaration of Helsinki. All of the study protocols were approved by the Research Ethics Committee of National Chengchi University Review Board.

### Stimuli and Task Design

All participants completed a word problem task during fMRI scanning. The task stimuli were compare word problems (Lewis and Mayer, 1987) constructed in traditional Chinese. Each problem consisted of three parts. The first part was an assignment statement expressing a variable (e.g., a dumpling costs 9 dollars). The second part was a relational statement denoting the value of another variable in relation to the predetermined variable (e.g., a wonton is 2 dollars less than a dumpling). The last part was a question asking about the quantity of the variable defined in the second part (e.g., how much does a wonton cost?). The relational statement varied across problems in its consistency and operation. The task comprised two sets of 80 compare word problems. For each set, half of the problems were consistent (the relational term was consistent with the operation, e.g., more—addition, whereas the other half were inconsistent such that the relational term did not match the operation, e.g., more—subtraction). For both consistent and inconsistent problems, 20 problems were selected for each operation, yielding a 2 (consistency) × 2 (operation) within-subject design. The correct solution plan of each word problem was a single-digit arithmetic task, with problems selected from any possible pairwise combinations of the digits between 2 and 9. Tie problems (e.g., 5 + 5) and problems containing 0 or 1 as an operand or the answer were excluded.

The first set of stimuli was administered during fMRI and presented in an event-related design (Figure 1). The whole set of 80 problems was broken up into four functional runs (i.e., 2 or 3 problems each condition per run). The sequence that each participant underwent the four runs was counterbalanced. Each trial began with a “+” sign as fixation for 500 ms, followed by a problem for 14 s to ensure participants had sufficient time to read through each problem, as determined from pilot testing results and evaluation of previous literature (Newman et al., 2011; Zhou et al., 2018; Chang et al., 2019). After the problem was presented, participants were instructed to choose the correct answer, *via* a button box, from three provided choice options: correct answer and two incorrect answers that were any two of ±1 of the correct answer, the reverse-operation error (i.e., calculating the sum of the two operands for subtraction or the difference of the two operands for addition problems), or ±1 of the reverse error within 2,750 ms. Afterward, the screen remained blank for a jittered intertrial interval between 2 and 5 s. Each run lasted ~7 min. The relatively long presentation of stimuli not only ensured that participants had enough time to read through the problems but also avoided motor responses contaminating the neural responses toward problem solutions, as the supplementary motor area (SMA) is consistently activated during arithmetic problem solving (Menon, 2015c). To obtain the actual behavioral performances, the other set of stimuli was used in a parallel task conducted outside the scanner using a self-paced procedure. Problem answers and distractors were presented simultaneously with each problem so that participants could respond as soon as they derive the answer. This out-of-scanner task was potentially more precise and replicable as it was conducted in a relatively normal environment with less potential for interference, and participants were more able to focus on problem solving at their own pace (Mencl et al., 2000; Chang et al., 2019).

### fMRI Data Acquisition

Neuroimaging data were acquired using a Siemens MAGNETOM Skyra 3-T scanner at National Chengchi University in Taipei City, Taiwan. Head movement was minimized during the scan by using cushions placed around the head of each participant. T2^*^-weighted echo-planar sequences were employed with the following parameters: repetition time (TR) = 2 s, time to echo (TE) = 30 ms, flip angle = 90, 36 interleaved axial slices with slice thickness = 4 mm, field of view = 220 × 220 mm^2^, matrix size = 64 × 64, providing an in-plane spatial resolution of 3.4 mm. In the same scan session, high-resolution T1-weighted anatomical scans were acquired using three-dimensional magnetization-prepared rapid-acquisition gradient-echo sequence for each participant to aid localization of functional data, with the following parameters: TR = 3,500 ms, TE = 3.37 ms, TI = 1,100 ms, flip angle = 7, field of view = 256 × 256 mm^2^, matrix size = 256 × 256, voxel size = 1 × 1 × 1 mm^3^, number of excitations = 1, 192 slices in the sagittal plane.

### fMRI Data Preprocessing

SPM12 (https://www.fil.ion.ucl.ac.uk/spm/software/spm12/) was used for the preprocessing of fMRI data. All functional images were corrected prior to statistical analysis for errors in slice timing, realigned to the first image of each run to correct for head motion, coregistered to each of the individual participant's structural scans, normalized to standard stereotaxic space (based on the Montreal Neurologic Institute coordinate system), and smoothed with a 6-mm full-width half-maximum Gaussian kernel to decrease spatial noise. No participants had motion >3 mm in the translational direction and 3 degrees in the rotational direction. The average movements of the final participants were 0.32 (SD = 0.15), 0.44 (SD = 0.20), and 0.76 (SD = 0.40) mm in the *x, y*, and *z* directions, with 0.78 (SD = 0.39), 0.28 (SD = 0.13), and 0.25 (SD = 0.12) degrees of roll, pitch, and yaw, respectively.

### Individual- and Group-Level Analysis

Statistical analysis was performed on both individual- and group-level data using the general linear model implemented in SPM12. Individual subject analyses were conducted by applying GLM that modeled the correctly responded trials as regressors and convolved with a canonical hemodynamic response function to model the expected BOLD signal. To account for individual differences in processing speed and to ensure each participant has sufficient time to read through each problem, each trial was modeled for the duration of each participant's mean response time plus one standard deviation from their out-scanner performance, with a maximum of 14 s. Incorrectly responded trials, the epoch participants made responses, and the six motion parameters generated in the realignment procedure of SPM12 were included as regressors of no interest. Voxel-wise *t* maps for each effect of interest from the individual level were then entered into a 2 (consistency) × 2 (operation) within-subject analysis of variance (ANOVA). We investigated the main effects and interactions at the brain level. Because *F* tests did not test the direction of the effects, *t* contrasts were calculated for visualization in the subsequent analyses to determine the direction of any significant effects. All significant results were determined according to a voxel-wise height threshold of *p* < 0.001 uncorrected and a multiple-comparison correction at a spatial-extent threshold of *p* < 0.05 family-wise error corrected after gray matter masking.

## Results

### Behavioral Performances

**Figure 2:**
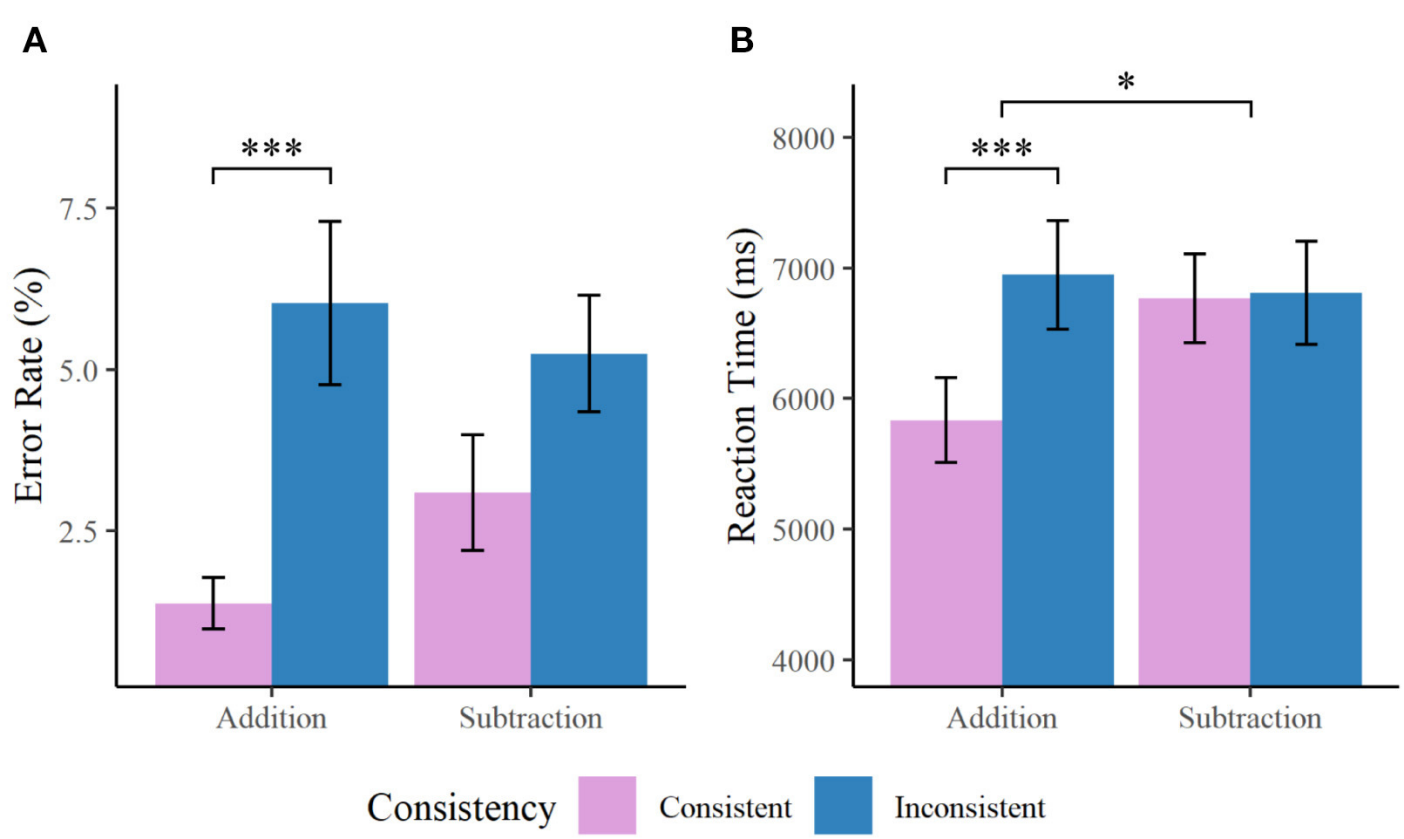
Bar charts showing **(A)** error rate and **(B)** reaction time of the out-of-scanner word problem task. Participants responded more accurately and faster to consistent than inconsistent problems. Interaction between consistency and operation was detected for the reaction time that lexically inconsistent problems were responded more slowly than consistent problems for addition, but such a difference was not observed for subtraction problems. *p < 0.05, ***p < 0.001.

As participants were not allowed to respond immediately after the presentation of word problems during the fMRI task, a different set of stimuli was used in a parallel task conducted outside the scanner using a self-paced procedure (for in-scanner behavioral results, see Supplementary Table 1). Further analyses on behavioral results were conducted based on the out-of-scanner task performance. Spearman rank correlation between error rate and response latency did not reach statistical significance, *r*_s_(32) = −0.24, *p* = 0.166, indicating no speed-accuracy tradeoff in the behavioral performance (Salthouse and Hedden, 2002). Mean error rate of each participant in each condition was entered into a 2 × 2 ANOVA involving consistency (consistent/inconsistent) and operation (addition/subtraction) as within-subject variables (Figure 2). The results revealed that participants responded less accurately to inconsistent than to consistent problems (error rate: 5.6 vs. 2.2%), *F*_(1, 33)_ = 15.14, *p* < 0.001, $\eta_{p}^{2}$ = 0.31. Difference in the error rate between addition and subtraction problems was not significant (3.7 vs. 4.2%), *F*_(1, 33)_ = 0.36, *p* = 0.552, $\eta_{p}^{2}$ = 0.01, nor was the consistency by operation interaction, *F*_(1, 33)_ = 2.37, *p* = 0.133, $\eta_{p}^{2}$ = 0.07.

Although adults' error rate of the task approached to the “floor,” and there was little variability in error rate across participants, the response time profile suggested that the task manipulation has sufficient loading to differentiate performance between distinct conditions. A parallel analysis was therefore conducted on the average reaction time of each participant to investigate the behavioral response profile (Figure 2). Both of the main effects were statistically significant, with participants responding more slowly to inconsistent than consistent problems (6,878 vs. 6,301 ms), *F*_(1, 33)_ = 17.47, *p* < 0.001, $\eta_{p}^{2}$ = 0.35, and subtraction more slowly than addition problems (6,788 vs. 6,391 ms), *F*_(1, 33)_ = 4.73, *p* = 0.037, $\eta_{p}^{2}$ = 0.13. The consistency by operation interaction was also significant, *F*_(1, 33)_ = 10.28, *p* = 0.003, $\eta_{p}^{2}$ = 0.24. In particular, participants responded more slowly for lexically inconsistent than consistent problems during addition, *F*_(1, 33)_ = 21.91, *p* < 0.001, $\eta_{p}^{2}$ = 0.40. For subtraction problems, however, such a difference in reaction time was not observed, *F*_(1, 33)_ = 0.05, *p* = 0.830, $\eta_{p}^{2}$ = 0.00. These results not merely suggested conventional operation effect and the expected consistency effect in arithmetic and word problem solving (Lewis and Mayer, 1987; Campbell and Xue, 2001; De Smedt et al., 2011; Rosenberg-Lee et al., 2011; Chang et al., 2015), but further implicated that the consistency effect was salient only during the correct problem model required addition. For subtraction, problems written in consistent and inconsistent format were likely equally difficult.

### Neuroimaging Results

#### Brain Responses Showed Minimal Difference While Solving Lexically Consistent and Inconsistent Word Problems

We first examined the brain regions that showed activation difference between consistent and inconsistent problems (Supplementary Table 2). We did not observe any regions within fronto-insular-parietal engagement that showed differences in brain responses between inconsistent and consistent problems. Rather, inconsistent problems elicited greater activations in the left lingual gyrus and the right cerebellum than consistent problems. In contrast, no significant clusters were detected for the contrast of consistent minus inconsistent problems.

#### Brain Responses Showed Minimal Difference Between Solving Word Problems Engaging Addition and Subtraction Problems

Next, we measured the difference in brain responses between addition and subtraction. The results showed that only one cluster showed greater activations during solving addition than subtraction problems. This cluster was lateral to the IPS and located in the right angular gyrus (Supplementary Table 3). By contrast, no significant activation was observed when contrasting subtraction over addition.

#### Brain Responses Showed Operation-Specific Lexical Consistency Effect

**Figure 3:**
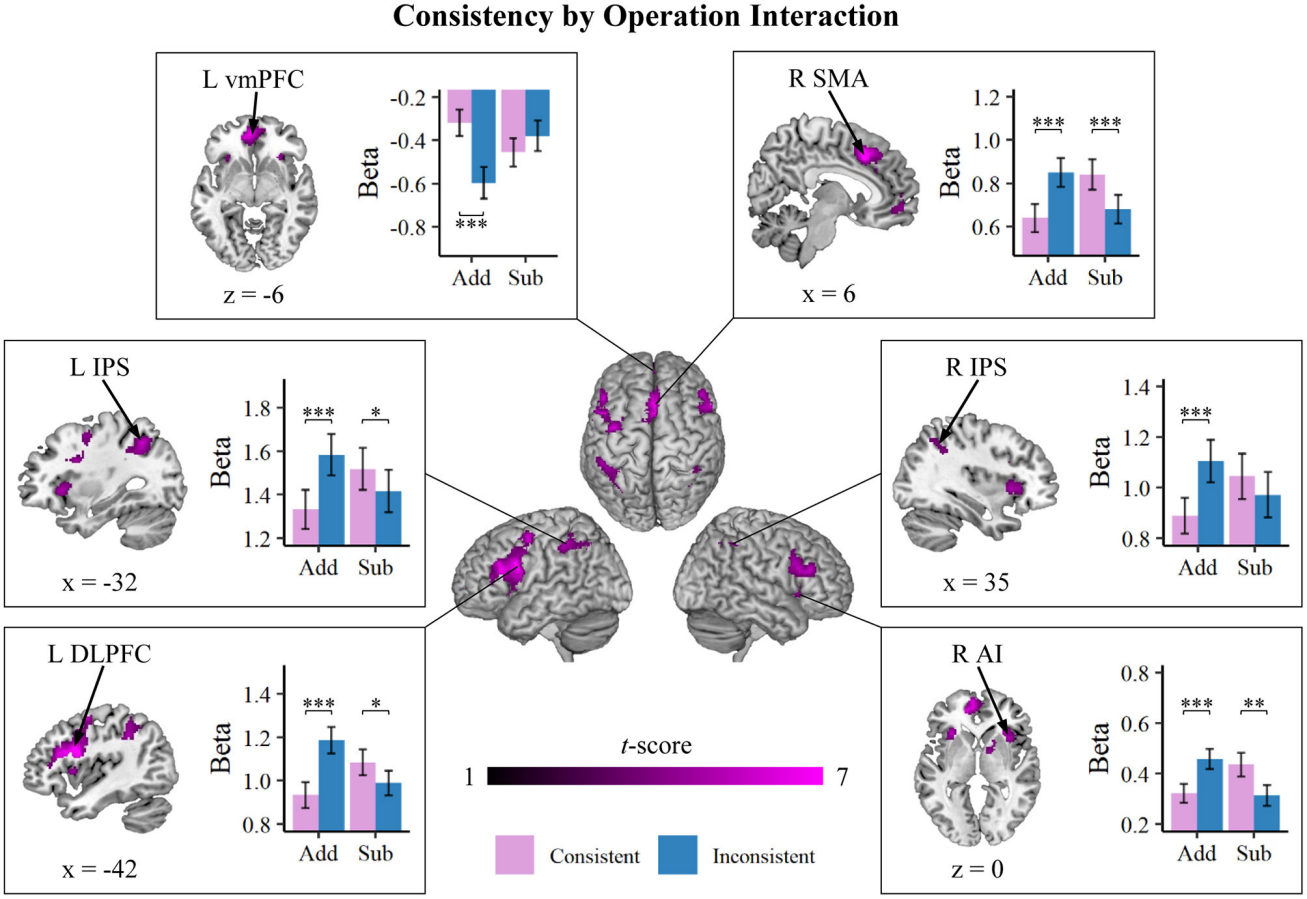
Brain regions that showed an interaction between consistency and operation. Inconsistent problems revealed stronger activations than consistent problems for addition (Add), whereas for subtraction (Sub), consistent problems were associated with greater activations than inconsistent problems. These effects manifested in the widespread fronto-insular-parietal network, including the anterior insula (AI), supplementary motor area (SMA), ventromedial prefrontal cortex (vmPFC), the bilateral dorsolateral prefrontal cortex (DLPFC), and the bilateral intraparietal sulcus (IPS). Error bars represent standard errors. *p < 0.05, **p < 0.01, ***p < 0.001.

We then investigated the interaction between consistency and operation, as shown in Table 2 and Figure 3. We identified a consistency by operation interaction within widespread brain regions, predominantly within the frontal-insular-parietal network. These regions included the bilateral IPS within the PPC, the bilateral DLPFC in the middle frontal gyrus (MFG), the SMA and the adjoining dACC, the right AI, the ventromedial prefrontal cortex (vmPFC) in the prefrontal cortex, and the caudate. *Post-hoc* simple-effect analyses further indicated that the interaction between consistency and operation was elicited from the lexically inconsistent problems showing stronger activations than the consistent problems during addition, primarily in bilateral MFG, SMA, dACC, right AI, and the IPS, and an inverse consistency effect during subtraction with consistent problems associated with greater activations than inconsistent problems. In the vmPFC, inconsistent problems revealed a stronger deactivation than consistent problems for addition, whereas no significant difference in deactivation was detected between consistency conditions for subtraction problems.

**Table 2:** Brain regions that showed the interaction of consistency by operation.

**Region**	**No. of voxels**	**Peak *t*-score**	**MNI coordinates**		
			***x***	***y***	***z***
*Addition > subtraction (inconsistent—consistent)*					
L dorsolateral prefrontal cortex/anterior insula	1959	8.24	−42	8	24
Bil supplementary motor area/dorsoanterior cingulate cortex	1153	7.74	6	20	42
R caudate	180	5.82	16	8	10
R anterior insula	247	5.73	30	26	0
L intraparietal sulcus	726	5.71	−32	−54	44
R dorsolateral prefrontal cortex	643	5.48	48	30	22
R intraparietal sulcus	146	4.06	36	−44	36
*Subtraction > addition (inconsistent*—*consistent)*					
L ventromedial prefrontal cortex	788	7.37	−12	48	−2

*L, left; R, right; Bil, bilateral*.

#### Operation-Specific Lexical Consistency Was More Salient in Less Proficient Problem Solvers

**Figure 4:**
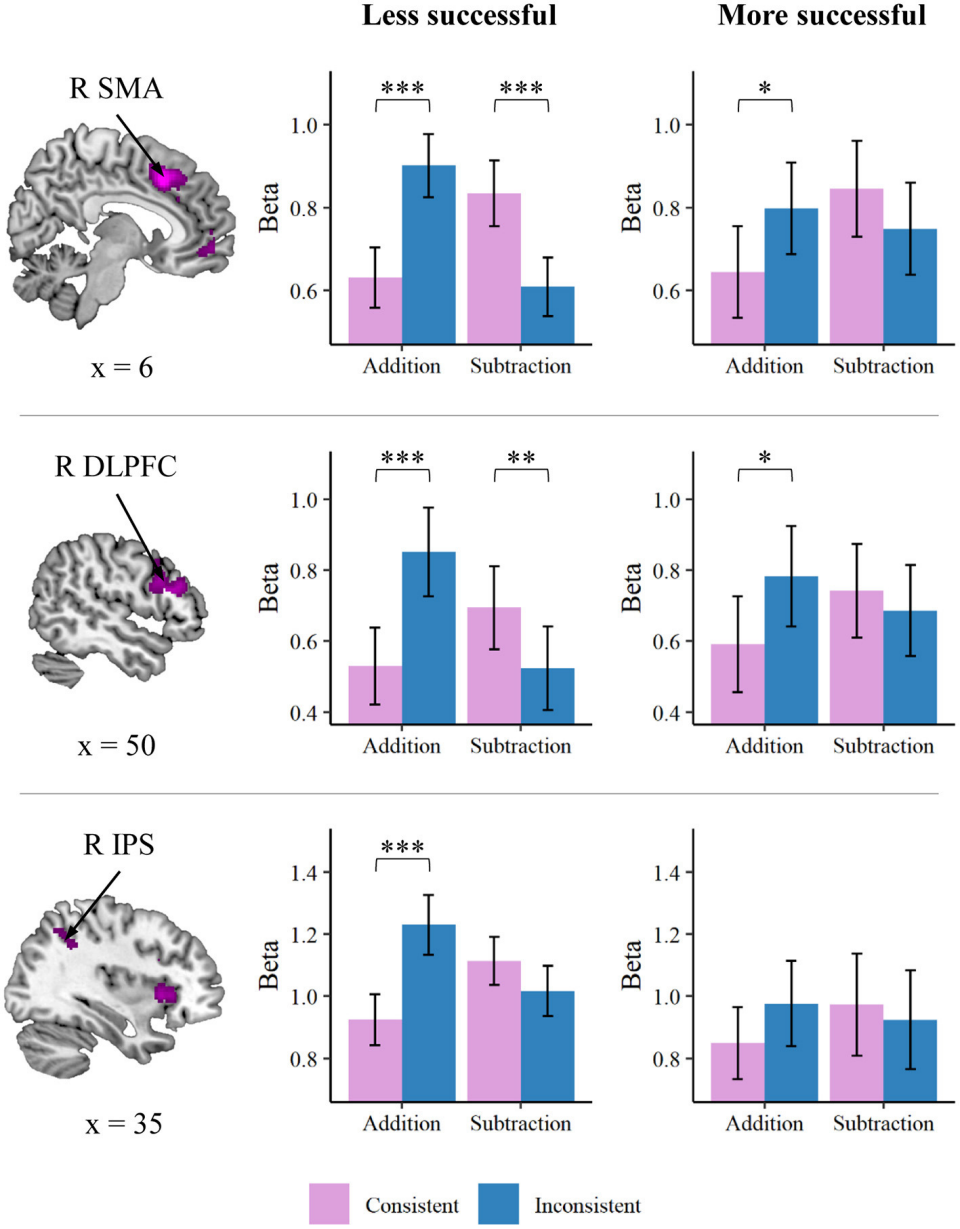
Brain regions that showed an interaction between group (more/less successful), consistency, and operation. The consistency by operation interaction was more prominent for the less successful than for the more successful word problem solvers in the supplementary motor area (SMA), right dorsolateral prefrontal cortex (DLPFC), and the right intraparietal sulcus (IPS). *p < 0.05, **p < 0.01, ***p < 0.001.

Because successful and unsuccessful problem solvers were considered as adopting different strategies (Hegarty et al., 1995), we then investigated brain-behavior association using a categorical approach to identify whether more and less successful problem solvers showed different response profiles of word problem solutions. To obtain a general performance index of word problem solution, we computed a composite measure of performance efficiency by averaging *z* scores for accuracy and negative *z* scores for reaction time for the overall word problem solving task (Salthouse and Hedden, 2002; Chang et al., 2015). Participants were then separated into either more or less successful word problem solver group based on the median of the efficiency score, resulting in 17 participants each group. This sample size was still beyond the estimated number of 15 per group for observing a three-way interactive effect with a power of 0.80 at α = 0.05, assuming a similar effect size of our previous study (Chang et al., 2019) as mentioned in the previous section. The average beta for each cluster identified from the consistency by operation interaction in each condition was entered into a mixed-design three-way ANOVA, with consistency and operation as within-subject factors and group (more/less successful) as a between-subject factor. As shown in Figure 4, the results indicated a three-way interaction in the SMA, *F*_(1, 32)_ = 6.43, *p* = 0.016, $\eta_{p}^{2}$ = 0.17, such that the interaction between consistency and operation was more prominent for the less successful word problem solvers, *F*_(1, 16)_ = 66.17, *p* < 0.001, $\eta_{p}^{2}$ = 0.81, than that for the more successful solvers, *F*_(1, 16)_ = 11.03, *p* = 0.004, $\eta_{p}^{2}$ = 0.41. There was marginal three-way interaction identified in the right DLPFC, *F*_(1, 32)_ = 3.73, *p* = 0.062, $\eta_{p}^{2}$ = 0.10, and the right IPS, *F*_(1, 32)_ = 3.15, *p* = 0.085, $\eta_{p}^{2}$ = 0.09. Likewise, the consistency by operation interaction appeared to be more salient for the less successful problem solvers than for the more successful solvers in both the two clusters: the right DLPFC: more successful solvers, *F*_(1, 16)_ = 8.80, *p* = 0.009, $\eta_{p}^{2}$ = 0.36; less successful solvers, *F*_(1, 16)_ = 26.96, *p* < 0.001, $\eta_{p}^{2}$ = 0.63; the right IPS: more successful solvers, *F*_(1, 16)_ = 3.24, *p* = 0.091, $\eta_{p}^{2}$ = 0.17; less successful solvers, *F*_(1, 16)_ = 24.58, *p* < 0.001, $\eta_{p}^{2}$ = 0.61. Differences in the interaction of consistency by operation between the more and less successful solvers for other clusters did not reach significance (*p* ≥ 0.145, $\eta_{p}^{2}$ ≤ 0.07).

## Discussion

In the current study, we investigated the neural circuits of how word problem deciphering varies between arithmetic operations in young adults that were mature in arithmetic skills. We presented compare problems that included relational terms and manipulated problem lexical consistency and operation of the problem solution plan. This design allowed us to investigate whether problem description affected the neural processing of numerical properties. Critically, we demonstrated a consistency by operation interaction such that when the correct solution plan of word problem required addition, problems presented with inconsistent language were associated with stronger brain activations than problems presented in consistent form, whereas the consistency effect was inverse when the operation was subtraction. Remarkably, this effect was observed in widespread fronto-insular-parietal regions important for cognitive control function during numerical problem solving (Menon, 2015c). Furthermore, problem solution proficiency was found to modulate these brain response profiles, with less successful problem solvers showing a more salient consistency by operation interaction. For the first time, this fMRI study demonstrates the lexical consistency of word problem reading is regulated by numerical processing at the neuronal level.

The central finding is that the effect of problem consistency on word problem solution is modulated by arithmetic operation, as manifested by the lexical consistency effect for addition problems and an inverse consistency effect for subtraction problems. This interaction effect washes out the main effect of either consistency or operation alone. According to our hypothesis, an interaction of consistency by operation would indicate supporting evidence that problems are solved *via* the intention to select numbers and relational terms that are primed by the arithmetic operation, whereas a main effect of problem operation would indicate problem solvers translate problem statement into the correct problem mathematical model regardless of the inherent markedness of the relational terms. Our results are in more favor of the former and have identified that the brain regions associated with word problem solutions vary with the relational terms. These results suggest that brain resources for solving arithmetic word problems are largely dependent on lexical marking of word problems, despite that problem performance is highly accurate.

The consistency by operation interaction is prominent in the distributed network encompassing AI, dACC, SMA, and MFG in prefrontal, as well as IPS in the posterior parietal regions. These circuits serve multiple cognitive functions necessary for successful numerical problem solving (Chang et al., 2015, 2016, 2019; Menon, 2015c). The AI coupling with dACC, DLPFC, and IPS form the crucial circuits that drive the cognitive control processes and guide the allocation of attention resources in the service of arithmetic problem solving (Menon, 2015c; Chang et al., 2019). Activations within the fronto-insular-parietal circuit generally reflect the increasing demands during more effortful arithmetic tasks, such as problems involving procedural calculation and manipulation of abstract quantity (Chochon et al., 1999; Grabner et al., 2009; De Smedt et al., 2011). It is expected that the fronto-insular-parietal circuits are more active during inconsistent than consistent problem. This is because problem solvers have a preference for the problem presentation order corresponding to consistent language problems (Lewis and Mayer, 1987). These results suggested that the non-preferred format of the word problem engaged extra loading of cognitive control process in the problem solution. Paradoxically, when the word problem solution required subtraction, it is consistent problems engaging stronger brain activations than when problems are presented in the inconsistent format.

How, then, do we understand the inverse consistency effect within fronto-insular-parietal circuits during subtraction? We suspect that these results reflect the solution of arithmetic word problems, with the relational term *less* (inconsistent addition and consistent subtraction) being more effortful and requiring greater calculation demand relative to *more* (consistent addition and inconsistent subtraction). These results have suggested that problem solvers apply the direct translation approach whereby it is the relational term rather than the solution plan that dominates problem solution (Lewis and Mayer, 1987; Hegarty et al., 1995; Boonen et al., 2016). Consistent with the lexical marking hypothesis that negative relational terms (e.g., less, smaller) appear to be more salient (Hegarty et al., 1992, 1995; Pape, 2003; Boonen et al., 2016), a higher level of the fronto-insular-parietal network associated numerical problem solving is required during processing negative relational terms. This is in stark contrast with the alternative hypothesis that the correct problem solution plan dominates the processing.

These results have further supported that problem solvers have a preference for problem presentation order, and the preferred order corresponds to consistent language (Lewis and Mayer, 1987). In one study conducted by Orrantia and Múñez (2013), participants were required to perform a figure discrimination task after reading a compare word problem sentence. They found that figure size discrimination performance is facilitated when the figure presented sequence matched the relational term (e.g., a larger figure preceded by a smaller figure coupled with the word problem of “John has 7 marbles more than Peter” denoting John > Peter). This suggests that participants automatically formulate a magnitude-based mental representation immediately based on word problem text. The generated mental representation of word problems can be linked to functional engagement of the fronto-insular-parietal circuits implicated in word problem solving (Chang et al., 2019), implicating the biological bases of word problem text decoding and that it can be initiated through neural functional modulation.

An additional explanation of the reversed consistency effect is that participants possibly solve inconsistent subtraction problems with indirect addition. Campbell (2008) has indicated that college students often solve subtraction problems (e.g., 9 − 5 = ?) through the corresponding addition (e.g., since 4 + 5 = 9 then 9 − 5 = 4). Subtraction problems presented in addition formats (e.g., 9 = ? + 5) can be advantageous over the standard formats (e.g., 9 − 5 = ?). An inconsistent subtraction problem such as “John has 9 marbles. John has 5 marbles more than Tom” would plausibly be represented as “9 = *T* + 5.” Such representation facilitates participants' usage of addition strategies for subtraction problem solution, thereby lowering the problem difficulty and resulting in the frontal-insular-parietal network activating to a lesser extent. In contrast, an inconsistent addition problem such as “Tom has 4 marbles. Tom has 5 marbles less than John” is likely represented as “4 = *T* − 5.” Consequently, it has to be further processed as “4 + 5 = *T*” before the final solution, and the additional step should increase calculation loading, thereby leading to an increased frontal-parietal activation (Prabhakaran et al., 2001). Further studies assessing problem solving strategies are needed to clarify this possibility.

We reveal that less successful problem solvers do not exhibit the same modulation within the fronto-insular-parietal network compared to more successful problem solvers. More specifically, the operation by consistency interaction is more salient in less successful problem solvers. Orrantia et al. (2015) demonstrated that there are response costs when operands of word problems are presented in number word over digit format. This format effect is more evident in low-achieving problem solvers. Hegarty and colleagues found that students with lower accuracy on word problem solving spent more time on initial direct translation of word problems and less time on problem integration and planning stage rather than high-accuracy students (Hegarty et al., 1992). In another study assessing eye movement during word problem solving, they found that as compared with successful problem solvers who made fewer errors, unsuccessful problem solvers spent more time on fixating numbers and relational terms (Hegarty et al., 1995). Together, these findings have indicated that individuals with low word problem solving skills are prone to superficially collecting numbers and relational keywords from problem descriptions and bypassing the correct problem solution models to a greater extent. These results offer educational implications from the neuronal level such that an effective strategy of instruction is likely to present problems that help students to avoid adopting direct translation.

In conclusion, our study provides evidence of neurobiological underpinnings for the brain representation of compare word problem solving, one of the most omnipresent and challenging pedagogical practices in the mathematics curriculum. Our findings show, for the first time, that problem description substantially alters neural mechanisms of how students process and comprehend arithmetic problems. Such neural characterization not only provides a scaffold for understanding cognitive components of strategies for mathematical learning but also likely contributes to promoting better strategies for remediating deficits and enhancing instructional design in the school curriculum. Our study helps to uncover the interplay of multiple functional circuits necessary for crucial cognitive skill acquisitions and speaks to parents and educators to pay attention to problem presentation materials of the school curriculum. Future studies can potentially benefit from investigating the developmental progression across critical learning stages of these mathematical practices.

## Data Availability Statement

The raw data supporting the conclusions of this article will be made available by the authors, without undue reservation.

## Ethics Statement

The studies involving human participants were reviewed and approved by National Chengchi University Review Board, Research Ethics Committee. The patients/participants provided their written informed consent to participate in this study.

## Author Contributions

C-TN analyzed the data and wrote the manuscript. T-CL designed the study, prepared stimuli, and collected the data. T-TC acquired funding, designed the study, monitored data collection and analyses, and wrote the manuscript. All authors approved the final version of the manuscript for submission.

## Conflict of Interest

The authors declare that the research was conducted in the absence of any commercial or financial relationships that could be construed as a potential conflict of interest.
