# Supplementary material for: Operation-Specific Lexical Consistency Effect in Fronto-Insular-Parietal Network During Word Problem Solving
Source: Front Hum Neurosci. 2021 Mar 10;15:631438. doi: 10.3389/fnhum.2021.631438 (PMC7987662; doi:10.3389/fnhum.2021.631438)
Supplement: Supplementary file 1 [file Table_1.DOCX]

*Supplementary Material*

# 1 Supplementary Tables

Table S1. Descriptive statistics of the behavioral data.

| Condition | | Error rate (%) | | | | Reaction time (s) | | | |
| --- | --- | --- | --- | --- | --- | --- | --- | --- | --- |
|  |  | Out-scanner | | In-scanner | | Out-scanner | | In-scanner | |
|  |  | *M* | *SD* | *M* | *SD* | *M* | *SD* | *M* | *SD* |
| Addition | Consistent | 1.4 | 2.3 | 1.8 | 2.7 | 5.83 | 1.89 | 0.80 | 0.12 |
|  | Inconsistent | 6.0 | 7.4 | 3.5 | 4.2 | 6.95 | 2.44 | 0.77 | 0.11 |
| Subtraction | Consistent | 3.1 | 5.2 | 3.1 | 4.1 | 6.77 | 1.99 | 0.77 | 0.11 |
|  | Inconsistent | 5.2 | 5.3 | 1.9 | 3.9 | 6.81 | 2.31 | 0.78 | 0.12 |

Table S2. Brain regions that showed differences between consistent and inconsistent problems.

| Region | | # of voxels | Peak *t*-score | MNI coordinates | | |
| --- | --- | --- | --- | --- | --- | --- |
|  |  |  |  | x | y | z |
| *Consistent > Inconsistent* | | | | | | |
|  | N/A |  |  |  |  |  |
| *Inconsistent > Consistent* | | | | | | |
|  | L Lingual Gyrus/ Middle Occipital Gyrus | 241 | 5.23 | –18 | –88 | 0 |
|  | R Cerebellum | 311 | 3.84 | 34 | –72 | –30 |

Table S3. Brain regions that showed response differences between addition and subtraction problems.

| Region | | # of voxels | Peak *t*-score | MNI coordinates | | |
| --- | --- | --- | --- | --- | --- | --- |
|  |  |  |  | x | y | z |
| *Addition > Subtraction* | | | | | | |
|  | R Angular Gyrus | 244 | 4.63 | 54 | –54 | 42 |
| *Subtraction > Addition* | | | | | | |
|  | NA |  |  |  |  |  |
